# Adjuvant Radiation Therapy in Breast Cancer Patients With Neurofibromatosis Type 1: Safety and Long-Term Outcomes

**DOI:** 10.1016/j.adro.2025.101931

**Published:** 2025-10-24

**Authors:** Carla Benmatallah, Youlia Kirova, Pierre Loap

**Affiliations:** a Department of Radiation Oncology, Institut Curie, Paris, France; b IHU des Cancers des Femmes, ANR, 23-IAHU-0006, Paris, France; c Université Versailles Saint Quentin (UVSQ), Versailles, France; d Proton Therapy Center (CPO), Institut Curie, Orsay, France; e Laboratoire d’Imagerie Translationnelle en Oncologie (LITO), Institut Curie, Orsay, France

## Abstract

**Purpose:**

Neurofibromatosis type 1 (NF1) is a rare autosomal dominant disorder associated with an increased risk of cancer, including a 5-fold higher incidence of breast cancer. Preclinical data suggest heightened radiosensitivity in NF1, raising concerns about toxicity and secondary malignancies after radiation therapy. Clinical data, however, are lacking. This study aimed to evaluate long-term outcomes and treatment-related toxicities in NF1 patients receiving adjuvant radiation therapy for breast cancer.

**Methods and Materials:**

This retrospective single-center study included 27 NF1 patients with nonmetastatic breast cancer treated with surgery and adjuvant radiation therapy between 1995 and 2023. Clinical, pathologic, treatment, and toxicity data were analyzed. Survival was assessed using Kaplan-Meier estimates, and toxicities were graded using the National Cancer Institute Common Terminology Criteria for Adverse Events.

**Results:**

Among 27 patients (30 tumors), the median follow-up was 79 months. The 10-year overall and cancer-specific survival rates were 68.3% each, and the metastasis-free survival rate was 68.4%. Local and locoregional control exceeded 92%. Acute radiodermatitis occurred in 83.3% of patients (grade 1-2); no grade ≥ 3 acute or late toxicity was observed. Late toxicities were rare and mild. No radiation-induced malignancy or cardiopulmonary events were reported. Nodal involvement and lymphovascular invasion were significantly associated with poorer survival.

**Conclusions:**

Adjuvant radiation therapy is safe and effective in NF1 patients with breast cancer, despite a notably high incidence of low-grade acute skin toxicity. No serious long-term toxicity or radiation-induced malignancy was observed. Modern dose-sparing techniques should be prioritized, and further prospective studies are needed to refine treatment approaches in this high-risk group.

## Introduction

Neurofibromatosis type 1 (NF1), or von Recklinghausen disease, is a rare autosomal dominant disorder affecting approximately 1 in 3000 live births. It results from inactivating mutations in the NF1 gene on chromosome 17, which encodes neurofibromin, a tumor suppressor that negatively regulates RAS/MAPK signaling.^1^ Loss of neurofibromin promotes uncontrolled cellular proliferation and tumor development. Individuals with NF1 have an increased predisposition to several malignancies, particularly malignant peripheral nerve sheath tumors,^2^, ^3^, ^4^ and face a 5-fold higher risk of breast cancer compared with the general population.^5^

Preclinical studies indicate that NF1-associated fibroblasts exhibit enhanced radiosensitivity,^6^, ^7^, ^8^, ^9^ raising concerns about an increased susceptibility to both acute and late radiation-induced toxicities. Moreover, NF1 patients may be at an elevated risk of secondary radiation-induced malignancies,^10^ including sarcomas.^3^

Adjuvant radiation therapy is a key component of breast cancer treatment, improving local control and survival following breast-conserving surgery or in node-positive patients after mastectomy.^11^^,^^12^ However, in NF1 patients, the therapeutic index of radiation therapy remains unclear. No clinical studies have specifically addressed its long-term safety or tolerability in this population. Given the underlying cellular radiosensitivity, there is a theoretical risk of increased normal tissue toxicity—cutaneous, cardiac, or pulmonary—as well as secondary cancers.^6^

To address this knowledge gap, we conducted a study to evaluate long-term outcomes and treatment-related toxicities of adjuvant radiation therapy in NF1 patients treated for breast cancer.

## Methods and Materials

### Study design and patient selection

This retrospective, single-center, observational study was conducted in the Department of Radiation Oncology of the Institut Curie (Paris, France). Eligible patients were adults with a confirmed diagnosis of NF1, established by either the National Institutes of Health clinical criteria or genetic testing, and histologically confirmed nonmetastatic breast cancer. Inclusion criteria required receipt of adjuvant radiation therapy following surgery. Patients with de novo metastatic disease were excluded.

Eligible cases were identified using a comprehensive text-mining search of the institutional electronic medical records. Data collection and analysis were performed in July 2025. The study was approved by the institutional review board of the Institut Curie and conducted in accordance with the Strengthening the Reporting of Observational Studies in Epidemiology (STROBE) guidelines for observational studies.

### Treatment characteristics

All patients underwent primary surgical management, either breast-conserving surgery or total mastectomy. Axillary staging was performed using sentinel lymph node biopsy or axillary dissection, as clinically indicated. Chemotherapy, administered in the neoadjuvant or adjuvant setting, and hormone therapy, in hormone receptor (HR)-positive cases, were delivered according to standard clinical guidelines.

Adjuvant radiation therapy was administered in accordance with institutional protocols. Whole-breast irradiation was performed following breast-conserving surgery. Chest wall irradiation was indicated after mastectomy in the presence of pathologically confirmed nodal involvement or adverse features (grade 3, triple-negative phenotype, or lymphovascular invasion). Regional nodal irradiation, including the supraclavicular (Berg’s level 4) and infraclavicular (axillary Berg’s levels 2-3) lymph node regions, was administered in cases of nodal involvement or high-risk medial tumors (T2 or larger, grade 3, triple-negative breast cancer [TNBC], lymphovascular invasion, or age < 40). In addition, axillary level 1 irradiation was permitted in cases of massive nodal involvement or positive sentinel lymph node biopsy without further dissection. The internal mammary chain could be irradiated at the discretion of the treating physician. A tumor bed boost was prescribed when appropriate (eg, age < 50, grade 3, TNBC, or lymphovascular invasion), delivered either sequentially or as a simultaneous integrated boost. Radiation therapy schedules included normofractionated or hypofractionated regimens, depending on the treatment era and clinical discretion.

Patients were treated in the supine position or in lateral isocentric decubitus (when nodal irradiation was not indicated), at the discretion of the treating radiation oncologist. Deep inspiration breath-hold (DIBH) was used when available to minimize cardiac and pulmonary exposure. All treatments were delivered using linear accelerators, employing 3-dimensional conformal radiation therapy, forward-planned field-in-field intensity modulated radiation therapy (IMRT), or volumetric modulated arc therapy (VMAT).

### Outcomes and statistical analysis

The primary endpoint was treatment-related toxicity, classified as acute (<6 months postradiation therapy) or late (≥6 months postradiation therapy), and graded according to the National Cancer Institute Common Terminology Criteria for Adverse Events. Toxicity assessments were recorded prospectively during routine follow-up. Secondary endpoints included overall survival (OS), cancer-specific survival (CSS), local recurrence-free survival (LRFS), locoregional recurrence-free survival (LRRFS), distant metastasis-free survival (MFS), and the incidence of radiation-induced malignancies. In patients with metachronous bilateral breast cancer, recurrence-free endpoints were analyzed per tumor, while OS and CSS were assessed per patient from the date of the first breast cancer diagnosis. Survival distributions were estimated using the Kaplan-Meier method.

Associations between clinical, pathologic, and treatment-related variables and survival or toxicity outcomes were assessed using Cox proportional hazards models and generalized linear models, respectively. Covariates included radiation therapy characteristics (dose, fractionation, target volumes, technique, and DIBH use), systemic therapy (chemotherapy), and tumor pathology (histologic subtype, TNM stage [restaged according to the American Joint Committee on Cancer (AJCC) Cancer Staging Manual, eighth edition], HR status, grade, and lymphovascular invasion). All statistical analyses were conducted using R software (version 4.1.0, R Foundation for Statistical Computing), with a 2-sided *P* value < .05 considered statistically significant.

## Results

### Patient and treatment characteristics

A total of 27 patients with NF1 and nonmetastatic breast cancer treated with surgery followed by adjuvant radiation therapy between January 1995 and December 2023 were included in this study. Among these, 3 patients developed metachronous bilateral breast cancer, resulting in a total of 30 distinct tumors. The median age at breast cancer diagnosis was 50 years (range, 37-81). The cohort was predominantly female (n = 26/27, 96.3%), with 1 male patient (n = 1/27, 3.7%). Two patients harbored additional pathogenic germline variants in cancer predisposition genes: 1 in RAD51C and 1 in PIK3CA. Across the cohort, 15 tumors were HR-positive/HER2-negative (50.0%), 2 were HR-positive/HER2-positive (6.7%), 4 were HR-negative/HER2-positive (13.3%), and 6 were TNBCs (20.0%). Complete receptor status was missing in 3 out of 30 tumors (10.0%), reflecting older data. The detailed clinicopathological features are presented in Table 1.

**Table 1:** Tumor characteristics of breast cancer patients with neurofibromatosis type 1 treated with adjuvant radiation therapy

Characteristics	No. of tumors (N = 30)	%
Age (y) at diagnosis, median (range)	50 (37-81)	-
Laterality		-
Left	15	50.00
Right	15	50.00
Histologic type		
IDC	26	86.67
Mixed invasive carcinoma	2	6.67
DCIS	2	6.67
cT		
Tis	2	6.67
T1	8	26.67
T2	16	53.33
T3	2	6.67
T4	2	6.67
cN		
N0	19	63.33
N+	11	36.67
Receptor status		
HR+/HER2−	15	50.00
HR+/HER2+	2	6.67
HR−/HER2+	4	13.33
TNBC	6	20.00
NA	3	10.00
Grade		
1	2	6.67
2	14	46.67
3	12	40.00
High grade (DCIS)	2	6.67
LVSI		
Present	11	36.67
Absent	15	50.00
NA	4	13.33

A total of 27 patients were included, including 3 with metachronous bilateral breast cancers, resulting in 30 distinct tumors.

*Abbreviations:* DCIS = ductal carcinoma in situ; HER2 = human epidermal growth factor receptor 2; HR = hormone receptor; IDC = invasive ductal carcinoma; LVSI = lymphovascular space invasion; NA = not assessable; TNBC = triple-negative breast cancer.

Two patients developed nonbreast malignancies during their lifetime. The first had a history of mediastinal neuroblastoma in childhood, treated with surgery and external beam radiation therapy. In adulthood, she was diagnosed with a left axillary neurofibrosarcoma treated with brachytherapy, both of which occurred prior to the diagnosis of the breast cancer included in this study. The second patient was diagnosed with endometrial carcinoma before her breast cancer treatment.

Treatment details are summarized in Table 2. Breast-conserving surgery was performed for 18 patients (n=18/30 tumors, 60.0%), though 2 later required complete mastectomy after radiation therapy, based on multidisciplinary board recommendations because of residual disease. Most patients received chemotherapy (n = 20/30, 66.7%), either in the neoadjuvant or adjuvant setting. Adjuvant radiation therapy was delivered to the intact breast in 18 patients and to the chest wall in 12 patients. Most treatments followed a normofractionated schedule, with a median total dose of 50.0 Gy (range, 32.5-51.8 Gy) administered in 25 fractions (range, 5-28) using 3-dimensional conformal techniques or forward-planned IMRT in the majority of cases (n = 26/30, 86.7%). Treatments were predominantly delivered under free-breathing conditions. Regional nodal irradiation was performed in 16 patients (53.3%), including the internal mammary chain in most of these cases (n = 14/16, 87.5%). Among the 18 patients treated with whole-breast irradiation, 11 (61.1%) received a tumor bed boost. Two of these patients underwent a simultaneous integrated boost, with prescribed doses of 63.0 Gy in 28 fractions and 64.4 Gy in 28 fractions.

**Table 2:** Treatment characteristics of breast cancer patients with neurofibromatosis type 1 treated with adjuvant radiation therapy

Treatment characteristics	No. of tumors (N = 30)	%
Surgery		
Primary treatment surgery		
Breast-conserving surgery	16	53.33
Total mastectomy	14	46.67
*Upfront*	*12*	*40.00*
*After BCT*	*2*	*6.67*
Axillary exploration		
Axillary dissection	23	76.67
SLNB	6	20.00
None (DCIS)	1	3.33
Chemotherapy		
Yes	20	66.67
*Neoadjuvant*	*11*	*36.67*
*Adjuvant*	*9*	*30.00*
No	10	33.33
Radiation therapy		
Volume		
Breast	18	60.00
Chest wall	12	40.00
Regional nodal irradiation		
Yes	16	53.33
*Inc. IMC*	*14*	*46.67*
*Inc. lower axillary area (Berg level 1)*	*1*	*3.33*
No	14	46.67
Boost		
Yes	11	36.67
*Sequential*	*9*	*30.00*
*SIB*	*2*	*6.67*
No	19	63.33
Technique		
3D-CRT/FiF-IMRT	26	86.67
VMAT	4	13.33
Positioning		
Dorsal decubitus	27	90.00
Lateral isocentric decubitus	3	10.00
Deep inspiration breath-hold		
Yes	2	6.67
No	28	93.33
Dose (Gy), median (range)	50.0 (32.5-51.8)	-
Fraction (n), median (range)	25.0 (5-28)	-

A total of 27 patients were included, including 3 with metachronous bilateral breast cancers, yielding 30 distinct tumors.

*Abbreviations:* 3D-CRT, 3-dimensional conformal radiation therapy; BCT = breast-conserving treatment; DCIS = ductal carcinoma in situ; FiF-IMRT, field-in-field intensity modulated radiation therapy; IMC = internal mammary chain; Inc = including; SIB = simultaneous integrated boost; SLNB = sentinel lymph node biopsy; VMAT = volumetric modulated arc therapy.

### Survival outcomes

**Figure 1:**
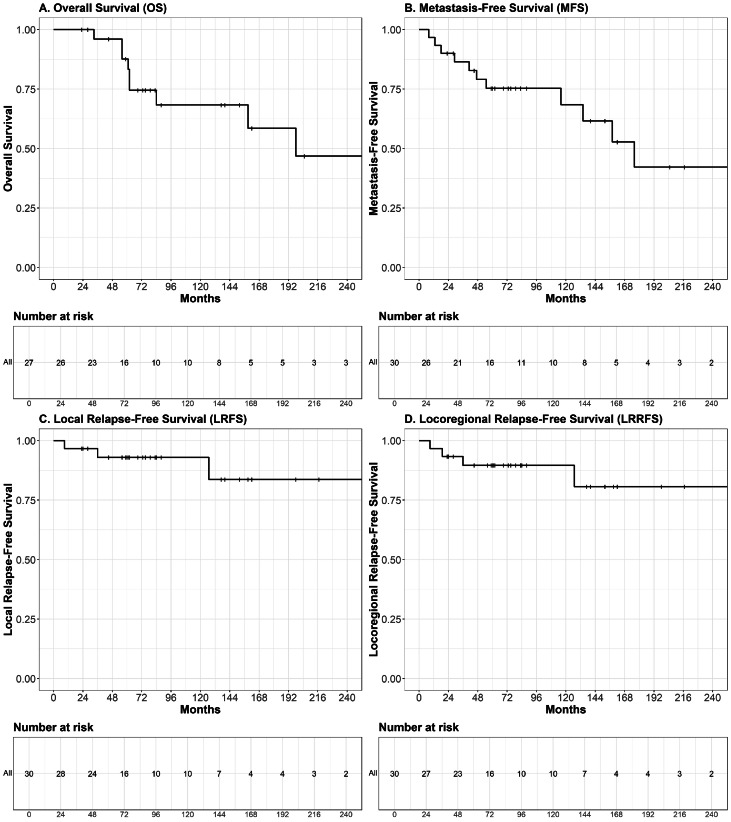
(A) Kaplan-Meier estimates of overall survival (OS), (B) metastasis-free survival (MFS), (C) local relapse-free survival (LRFS), and (D) locoregional relapse-free survival (LRRFS) in breast cancer patients with neurofibromatosis type 1 treated with adjuvant radiation therapy (N = 27). For patients with metachronous bilateral breast cancer (n = 3), recurrence-free endpoints were analyzed per tumor, while OS was calculated per patient from the date of the first breast cancer diagnosis.

At the time of analysis, the median follow-up time was 79 months per patient (range, 23-363) and 77 months per tumor (range, 23-363). During this period, 9 patients died, including 8 from breast cancer. The median OS was 198 months (95% CI, 159-not reached), and the median MFS was 176 months (95% CI, 134-not reached). The median CSS, LRFS, and LRRFS were not reached. Kaplan-Meier curves for OS, MFS, LRFS, and LRRFS are presented in Fig. 1.

OS and CSS were both estimated at 87.7% (95% CI, 75.5%-100%) at 5 years, and 68.3% (95% CI, 51.0%-91.5%) at 10 years. MFS was 75.3% (95% CI, 60.9%-93.1%) at 5 years and 68.4% (95% CI, 51.6%-90.8%) at 10 years. LRFS was 92.9% (95% CI, 84.0%-100%) and LRRFS was 92.4% (95% CI, 82.9%-100%) at both time points. Three local recurrences were observed, occurring at 9, 36, and 127 months after initial diagnosis, respectively. All were diagnosed in patients under the age of 50 (*P* = .04), and exclusively among those who had undergone breast-conserving surgery (*P* < .03). These tumors were of low to intermediate grade (1 grade 1 and 2 grade 2). They included 1 TNBC and 1 HER2-positive tumor, while the third had unknown receptor status, having been diagnosed in 1995. One isolated axillary nodal recurrence was observed 19 months after diagnosis in a patient aged <50 years who had undergone total mastectomy for a grade 2, HER2-positive, HR-negative, cT3N1 tumor.

**Figure 2:**
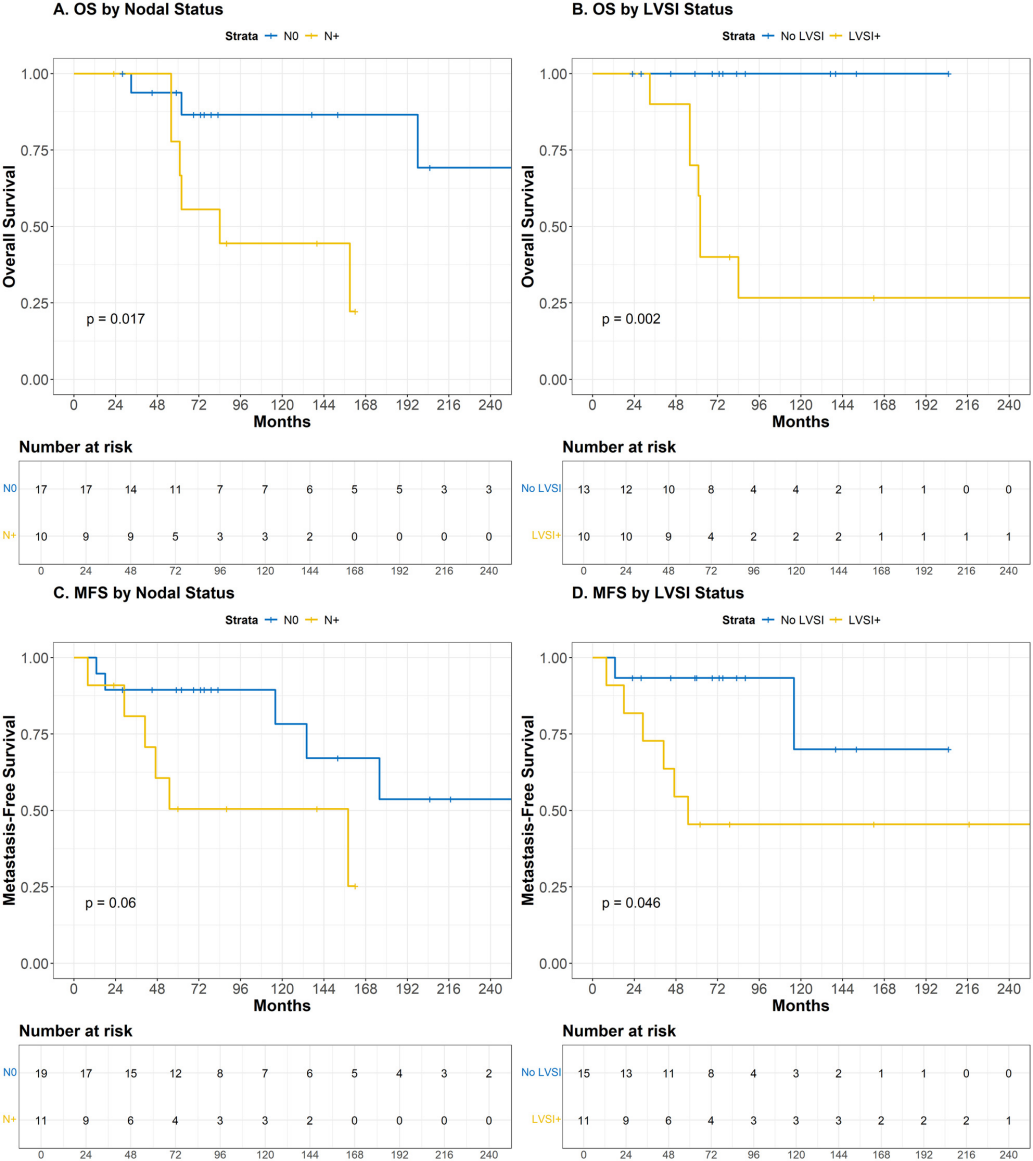
Kaplan-Meier estimates of overall survival (OS) and metastasis-free survival (MFS) stratified by (A and B) nodal status and (C and D) lymphovascular space invasion (LVSI) in breast cancer patients with neurofibromatosis type 1 treated with adjuvant radiation therapy. In patients with metachronous bilateral breast cancer (n = 3), recurrence-free outcomes were analyzed per tumor, while OS was calculated per patient from the date of the first breast cancer diagnosis.

Nodal involvement was statistically associated with both OS and CSS (*P* = .02 for each), and showed a trend toward significance with MFS (*P* = .060). Similarly, the presence of lymphovascular space invasion (LVSI) was significantly associated with poorer OS (*P* < .01), CSS (*P* < .01), and MFS (*P* = .046). In patients with nodal involvement, 10-year OS and MFS were 44.4% (95% CI, 21.4%-92.3%) and 45.0% (95% CI, 21.8%-92.7%), respectively, compared with 86.5% (95% CI, 70.7%-100%) and 74.9% (95% CI, 52.5%-100%) in node-negative patients (Fig. 2). In the presence of LVSI, 10-year OS and MFS were 26.7% (95% CI, 8.9%-80.4%) and 30.0% (95% CI, 11.6%-77.3%), respectively, compared with 100% and 75.0% (95% CI, 42.6%-100%) in LVSI-negative patients (Fig. 2). The small number of locoregional events limited univariate analyses of LRFS and LRRFS.

### Toxicity outcomes

Radiation-related toxicity following breast irradiation was generally low across the cohort (Table 3). No grade ≥ 3 acute or late toxicities were observed. The most common acute adverse event was radiodermatitis, occurring in 25 patients (83.3%), with grade 1 in 20 cases (66.7%) and grade 2 in 5 (16.7%).

**Table 3:** Radiation-related toxicity in breast cancer patients with neurofibromatosis type 1 treated with adjuvant radiation therapy

	Grade (CTCAE), N = 30 n (%)			
Toxicity type	1	2	3	4-5
Acute toxicity				
Radiodermatitis	20 (66.67)	5 (16.67)	0	0
Breast edema	4 (13.33)	0	0	0
Late toxicity				
Radiodermatitis	4 (13.33)	0	0	0
Breast fibrosis	1 (3.33)	1 (3.33)	0	0
Breast edema	1 (3.33)	0	0	0
Pulmonary fibrosis	2 (6.67)	0	0	0

*Abbreviations:* CTCAE = National Cancer Institute Common Terminology Criteria for Adverse Events.

Late toxicities were infrequent and mild, with the highest grade corresponding to a single case of grade 2 breast fibrosis. Two cases of late radiographic pulmonary fibrosis (grade 1, 6.7%) were also documented. One patient, who had received mediastinal irradiation at the age of 8 for a childhood mediastinal neuroblastoma (1958, 2-dimensional technique), later developed a clinically evident left pulmonary fibrosis (grade 2) within the previously irradiated field, along with right-sided heart failure. These complications were primarily attributed to prior thoracic irradiation rather than to right-sided adjuvant breast radiation therapy. Accordingly, no clinical cardiopulmonary toxicity was directly linked to breast irradiation. No radiation-induced malignancy or secondary sarcoma was observed in the cohort.

There was no association between the occurrence of toxicity and patient age, radiation therapy technique (3-dimensional conformal radiation therapy vs IMRT), or fractionation schedule (normofractionated vs hypofractionated).

## Discussion

To our knowledge, this is the first published cohort specifically investigating the use of adjuvant radiation therapy in patients with NF1 treated for nonmetastatic breast cancer. Despite theoretical concerns about increased radiosensitivity in this population, our findings suggest that adjuvant breast radiation therapy is both feasible and well tolerated in NF1 patients, with excellent local control and a favorable toxicity profile.

In our NF1 cohort, locoregional control was excellent, with LRFS and LRRFS exceeding 92% at both 5 and 10 years. These results are in line with those from the phase 3 HypoG-01 trial,^13^ in which 5-year LRFS reached approximately 92% to 93% in both hypofractionated and normofractionated arms. The concordance of the outcomes suggests that NF1 patients achieve locoregional control rates comparable with those observed in the general breast cancer population when treated with adjuvant radiation therapy.

To date, evidence on radiation therapy tolerance in NF1 breast cancer patients is limited to a single case report describing a 66-year-old woman treated with breast-conserving surgery, radiation therapy (40.5 Gy), and hormone therapy, with no recurrence or sarcoma at 2 years.^14^ However, no details were reported regarding acute or late toxicity.

The majority of our patients experienced mild acute toxicity, predominantly grade 1 to 2 radiodermatitis. No grade ≥ 3 acute or late toxicities were observed. Although the incidence of acute skin toxicity appeared twice as high as what is typically reported in large prospective cohorts, such as CANcer TOxicities (CANTO),^15^, ^16^, ^17^ this increased rate did not translate into significant long-term sequelae. Importantly, only 1 patient developed grade 2 breast fibrosis. These findings are reassuring and support the safety of adjuvant radiation therapy in this genetically predisposed population. It is worth noting that while the increased incidence of acute radiodermatitis may not have led to clinically significant long-term consequences, it could affect patient quality of life and psychological well-being. Patients should be informed of this potential side effect during treatment planning. The use of modern delivery techniques, such as VMAT^15^ and hypofractionation schedules,^18^, ^19^, ^20^ can help mitigate this risk and should be considered standard when available.

Pulmonary toxicity was rare and generally mild. Two patients developed asymptomatic grade 1 radiographic pulmonary fibrosis, likely attributable to breast irradiation. A third patient, who had received large-field mediastinal irradiation in childhood for a neuroblastoma, later developed left-sided pulmonary fibrosis and right heart failure in adulthood. Given the field geometry and laterality, these events were attributed to prior thoracic irradiation rather than to radiation therapy for her right breast cancer. Across the cohort, no cases of radiation-induced secondary malignancies or sarcomas were observed, which is particularly reassuring given the long follow-up and theoretical susceptibility of NF1 patients to radiation-induced tumors.

Treatment-related cardiac toxicity was not observed in our study, but it remains a potential concern in this population.^21^ NF1 patients tend to be diagnosed at a younger age because of their elevated lifetime risk of breast cancer, resulting in longer posttreatment survival and prolonged exposure to potential late effects, including those of systemic therapies^22^, ^23^, ^24^; critical cardiac substructure sparing^25^, ^26^, ^27^, ^28^ is therefore essential. Although only a minority of patients in our cohort were treated with modern techniques, such as VMAT or DIBH,^22^^,^^28^^,^^29^ these strategies have proven effective in minimizing cardiac exposure and should be strongly considered. Proton therapy may also be advantageous in selected cases,^30^, ^31^, ^32^, ^33^ particularly in patients with known radiosensitivity mutations (eg, ATM and TP53),^34^ because it enables a significant reduction in both cardiac dose^30^ and low-dose bath to surrounding tissues. Techniques such as isocentric lateral decubitus may also contribute to dose-sparing^35^ in patients who do not require nodal irradiation.

The choice of fractionation remains an open question in NF1 patients, because no prospective data support the oncologic equivalence of hypofractionation in this specific population. Nevertheless, in our cohort, the use of hypofractionated schedules was not associated with increased toxicity or reduced efficacy. Given the low recurrence rate observed and the radiobiological advantages of hypofractionation in reducing the cumulative exposure to organs at risk, such as the heart,^36^^,^^37^ it appears reasonable to adopt moderate hypofractionation, including nodal irradiation, when clinically appropriate.^13^^,^^38^ This approach may be particularly beneficial in patients who have received or will receive cardiotoxic chemotherapy such as anthracyclines.

From a clinical perspective, our findings do not support omitting adjuvant radiation therapy in NF1 patients, because treatment was generally well tolerated without excess acute or late toxicity. Standard indications for radiation therapy should therefore be maintained. Nevertheless, given the theoretical risk of radiation-induced sarcomas in this genetically predisposed population, dose-sparing strategies deserve particular attention. Partial breast irradiation may be an option in carefully selected early-stage patients to reduce exposure of uninvolved tissues.^39^^,^^40^ Similarly, proton therapy, by substantially lowering the integral dose and improving lung sparing, may be particularly relevant in younger NF1 patients who face a longer life expectancy and higher cumulative risks of secondary malignancy. While our data do not show increased clinical radiosensitivity, these theoretical considerations highlight the need for caution and justify the use of modern, highly conformal techniques whenever available.

Despite the inherent limitations related to sample size, the principal strength of this study lies in its novelty, representing the first detailed clinical assessment of radiation therapy outcomes in patients with NF1-associated breast cancer. The consistency of the observed results, combined with long-term follow-up, offers meaningful preliminary evidence to inform future treatment strategies in this high-risk population.

## Conclusions

This study represents the first clinical evaluation of adjuvant radiation therapy in patients with NF1 treated for nonmetastatic breast cancer. Our findings suggest that adjuvant radiation therapy is feasible and generally safe in this population, with excellent locoregional control and no evidence of severe late toxicity or secondary malignancies. However, the incidence of acute radiodermatitis was notably high, affecting more than 80% of patients, though limited to grade 1 to 2 severity. While this may reflect increased radiosensitivity in NF1 patients, these toxicities were transient and did not lead to lasting sequelae. Therefore, NF1 should not be considered a contraindication to standard adjuvant radiation therapy. Whenever possible, modern radiation techniques and dose-sparing strategies—such as hypofractionation, VMAT, DIBH, or proton therapy—should be used, particularly in younger patients. Further prospective research is needed to optimize treatment and confirm these preliminary findings.

## Disclosures

The authors declare that they have no known competing financial interests or personal relationships that could have appeared to influence the work reported in this paper.
